# Promotion of biological nitrogen fixation activity of an anaerobic consortium using humin as an extracellular electron mediator

**DOI:** 10.1038/s41598-021-85955-3

**Published:** 2021-03-22

**Authors:** Sujan Dey, Takanori Awata, Jumpei Mitsushita, Dongdong Zhang, Takuya Kasai, Norihisa Matsuura, Arata Katayama

**Affiliations:** 1 Graduate School of Engineering, Nagoya University, Chikusa-ku, Nagoya, 464-8603 Japan; 2 National Institute for Land and Infrastructure Management, Asahi 1, Tsukuba, Ibaraki 305-0804 Japan; 3 Institute of Materials and Systems for Sustainability, Nagoya University, Chikusa-ku, Nagoya, 464-8603 Japan; 4 School of Geosciences and Civil Engineering, Kanazawa University, Kakuma-machi, Kanazawa, Ishikawa 920-1192 Japan; 5 Present Address: Ocean College, Zhejiang University, Zhoushan, 316021 China

**Keywords:** Environmental microbiology, Environmental biotechnology

## Abstract

Nitrogen fertiliser is manufactured using the industrial Haber–Bosch process, although it is extremely energy-consuming. One sustainable alternative technology is the electrochemical promotion of biological nitrogen fixation (BNF). This study reports the promotion of BNF activity of anaerobic microbial consortia by humin, a solid-phase humic substance, at any pH, functioning as an extracellular electron mediator, to levels of 5.7–11.8 times under nitrogen-deficient conditions. This was evidenced by increased acetylene reduction activity and total nitrogen content of the consortia. Various humins from different origins promoted anaerobic BNF activity, although the degree of promotion differed. The promotion effected by humin differed from the effects of chemical reducing agents and the effects of supplemental micronutrients and vitamins. The promotion of anaerobic BNF activity by only reduced humin without any other electron donor suggested that humin did not serve as organic carbon source but as extracellular electron mediator, for electron donation to the nitrogen-fixing microorganisms. The next generation sequencing (NGS) of partial 16S rRNA genes showed the predominance of Clostridiales (Firmicutes) in the consortia. These findings suggest the effectiveness of humin as a solid-phase extracellular electron mediator for the promotion of anaerobic BNF activity, potentially to serve for the basis for a sustainable technology.

## Introduction

Fixation of atmospheric nitrogen to ammonia is crucial to maintain the global nitrogen cycle and provide N fertiliser for the growth of plants, including crops. At present, the majority of nitrogen fertilisers are produced by the Haber–Bosch process^1^. However, ammonia production by the Haber–Bosch process is extremely energy-consuming, utilising 1–2% of total world energy-use and releasing approximately 2.5% of global CO_2_ emissions^2,3^. Biological nitrogen fixation (BNF) is expected to become an alternative to the Haber–Bosch process as a means of ammonia production because of its lower energy consumption. For the application of BNF to produce ammonia, high activity of nitrogen-fixing microorganisms is essential because of the slow kinetics of biological production of ammonia^4^.

The process of BNF is carried out by diazotrophs, using nitrogenase, an enzyme catalysing the biological N-reducing reaction (Eq. 1).

1 $${\text{N}}_{{2}} + {\text{8H}}^{ + } + {\text{8e}}^{ - } + {\text{16ATP}} \to {\text{2NH}}_{{3}} + {\text{H}}_{{2}} + {\text{16ADP}} + {\text{16Pi}}$$

Nitrogenase is a complex oxidoreductase that is classified into three different types based on the heterometal cofactor present in the active site of the enzyme complex^5,6^. There is a wide range of diazotrophs, including free living (consisting of aerobic, microaerophilic, and anaerobic) and symbiotic bacteria, cyanobacteria, and archaea. Some species of *Geobacter*, for example *G. sulfurreducens* and *G. metallireducens*, have also been reported as N-fixers^7,8^. These broadly distributed diazotrophs fix atmospheric nitrogen under complex and specific conditions, including the type of host plants and a range of environmental factors.

In recent years, efforts have been made to increase the activity of nitrogenase by direct input of electrons: electrocatalysis^9^, bioelectrocatalysis using immobilised nitrogenase^10^, photocatalysis^11–15^, and nitrogen reduction by transition metal catalysis mimicking nitrogenase^16–18^. These have been devoted to finding efficient alternatives for the Haber–Bosch process to synthesise ammonia under ambient conditions. However, in these approaches, limitations arise from low conversion efficiencies, poor selectivity and expensive catalysts^19^. Moreover, irreversible damage to nitrogenase occurs when it is exposed to O_2_ through immobilisation, making bioelectrocatalysis untenable for N reduction. Nitrogenase loosed the activity also by changes in the conditions such as pH. In addition, the ammonia synthesised in these approaches must be converted to fertiliser to make it available for plants, another energy-consuming chemical reaction. These makes the use of purified enzymes more expensive and difficult in handlings, compared to self-regulated intact cell^20^. Therefore, promotion of BNF in living, intact, microorganisms, rather than nitrogen fixation of purified enzymes, can be a more sustainable approach to the Haber–Bosch process. Because the reaction rates of BNF are dependent on energy for dinitrogen reduction to ammonia, the supply of electrons to nitrogen-fixing microorganisms through extracellular electron mediators may increase the efficiency of the BNF reaction (Eq. 1), thus accelerating BNF.

Recently, various extracellular electron mediators including humic substances and artificial electron mediators have been reported to accelerate different microbial reactions in relation with carbon and nitrogen cycles^21–27^. Some artificial electron mediators, methyl viologen and other viologens, have been used in dinitrogen fixation for the bio-electrochemical synthesis of ammonia^28^. However, methyl viologen and other viologens are water soluble, toxic to the environment, and human health^29^. Water-soluble and toxic extracellular electron mediators can be washed away from the reactor and into the environment, decreasing the applicability of the mediator. Consequently, insoluble, chemically stable, non-toxic materials should be candidates as extracellular electron mediators for promoting microbial processes. Humin is an environmentally harmless organo-mineral humic substance extracted from soil, insoluble in any pH conditions, formed by the decomposition of organic materials. Currently, humin has been reported as an extracellular electron mediator for different microbial reducing reactions, including reductive dehalogenation^30,31^, nitrate reduction to ammonia, iron reduction^32^, and denitrification^33^. Although humin has advantageous characteristics as an extracellular electron mediator, including its stability in nature and no loss in its application, the effect of humin on BNF has not been reported to-date.

Thus, the objective of this study is to examine if the BNF activity/efficiency of anaerobic consortia can be promoted by supplying extracellular electrons through an extracellular electron mediator-humin.

## Results

### Promotion of BNF activity in anaerobic consortia by humin

**Figure 1:**
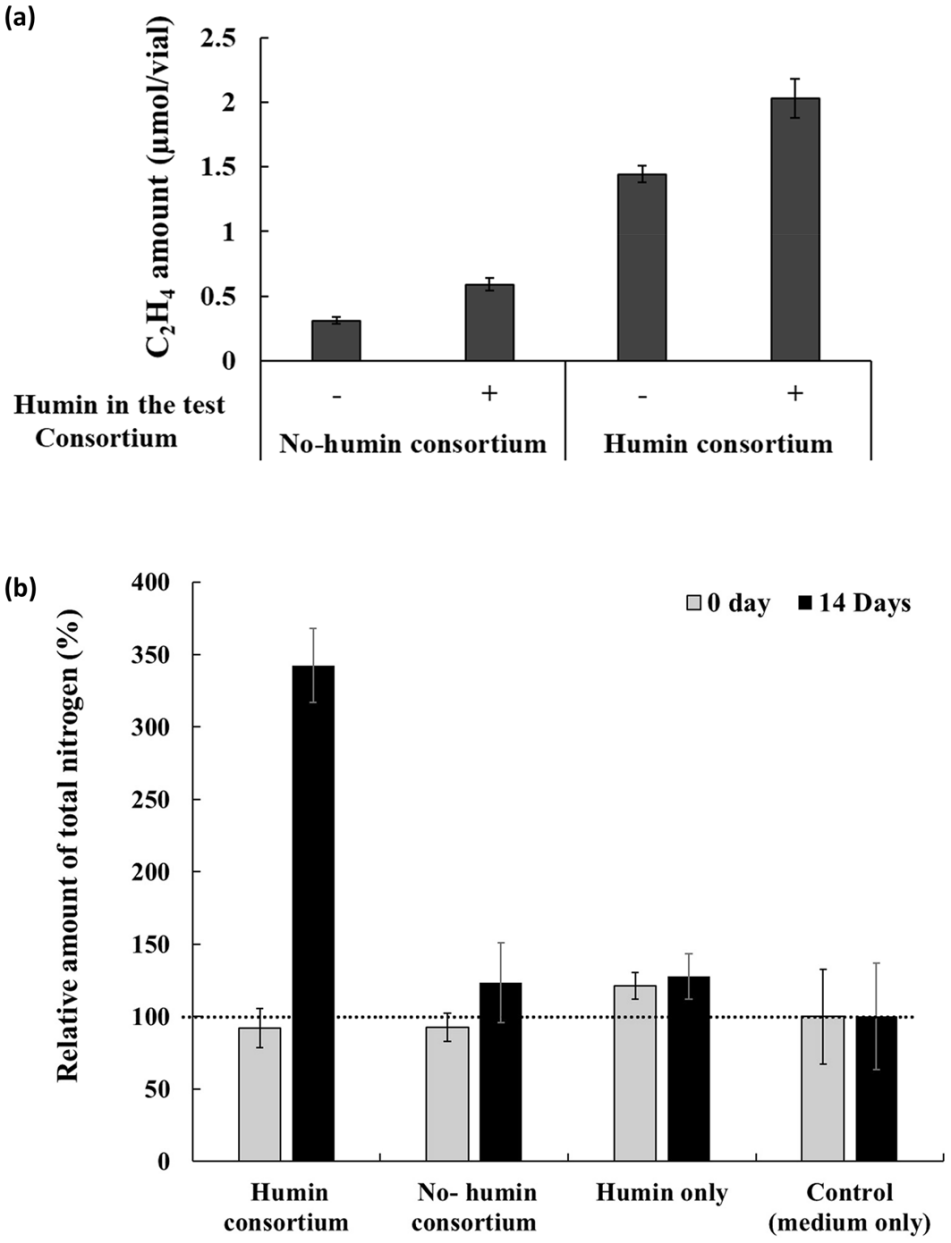
(**a**) ARA of the humin and no-humin consortia using testing conditions with (+) and without humin (−); (**b**) changes in the relative total nitrogen content in the cultures under four conditions during 14 days of incubation: humin consortium, no-humin consortium, humin only and control (medium only, as 100%). The humin used here was Kamajima humin.

Two nitrogen-fixing anaerobic microbial consortia were enriched from Kamajima paddy soil by transfer using an anaerobic, modified nitrogen-deficient, Ashby medium (anaerobic MNDA medium) with and without supplementation of humin every two weeks (herein referred to as humin consortium and no-humin consortium, respectively). Anaerobic BNF activity of the consortia was determined using both acetylene reduction activity (ARA) and the increase in nitrogen content in the consortia. The enriched consortia exhibited stable ARA after the 11th generation of enrichment (Fig. S1). Thus, consortia after the 11th generation were subjected to the experiments in this study. Figure 1a shows the effect of humin on ARA in the consortium. The humin consortium exhibited higher ARA than the no-humin consortium, regardless of the ARA test conditions with and without humin. In addition, both consortia showed higher ARA under test conditions with humin than those without humin. The promotion of anaerobic BNF activity was also confirmed by the increase in total N in the consortia after incubation at 30 °C for 14 days (Fig. 1b). Extensive promotion of anaerobic BNF activity was observed in the humin consortium (342% to the control) compared with the no-humin consortium (127% to the control). No increase was observed in humin only (without inoculum), indicating no chemical nitrogen fixation. These results clearly suggest that the promotion of anaerobic BNF activity in the humin consortium required both the consortium and humin. It should be noted that anaerobic BNF activity was promoted by humin beyond the level observed under nitrogen-deficient conditions. Figure 1a shows that humin addition promoted ARA by 90% for no-humin consortium and by 40% for humin consortium in the test. In comparison between the no-humin consortium tested without humin and the humin consortium with humin, ARA was higher by 6.7 times in the latter. Based on the relative amount of total nitrogen (Fig. 1b), counting the nitrogen fixation in the no-humin consortium (27%) and the contribution of nitrogen content of the humin itself (23%), the humin consortium promoted the BNF reaction by 292%, 11.8 times of the no-humin consortium, under nitrogen-deficient conditions.

### Ubiquitous promotion effect of Humin on anaerobic BNF activity

**Figure 2:**
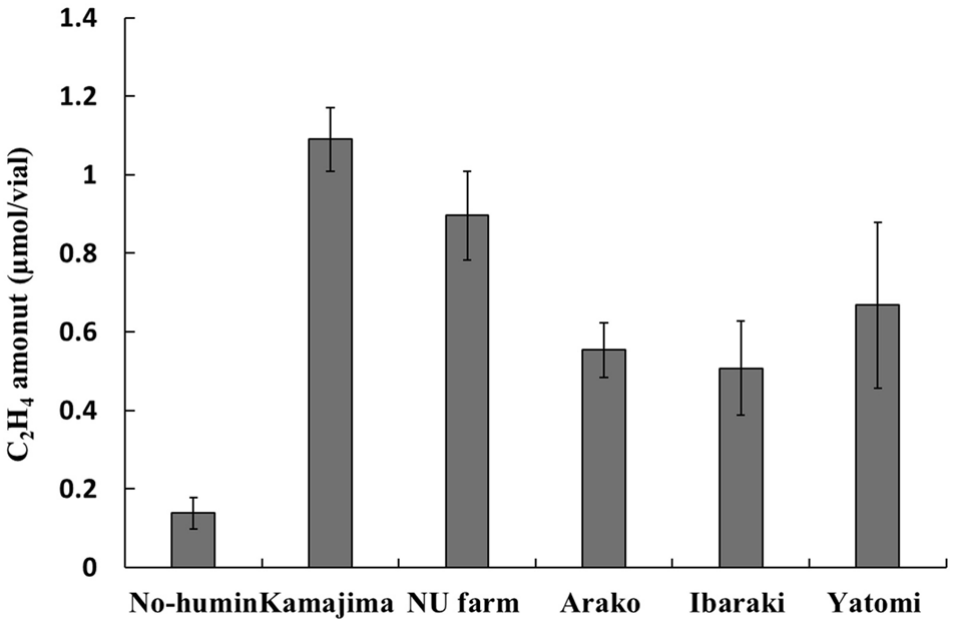
Effects of various humins on ARA of the no-humin consortium. All humins examined promoted ARA of the no-humin consortium. Kamajima humin yielded the highest ARA in the no-humin consortium.

Effects of various humins extracted from different source soils and sediment were examined using the ARA test applied to the no-humin consortium (Fig. 2). All humins significantly promoted the ARA of the no-humin consortium compared with the no-humin consortium without humin. Among the five humins, the largest promotion was observed in the consortium with Kamajima humin, extracted from Kamajima paddy soil. Therefore, Kamajima humin was used for further studies.

### Effect of redox state of humin on anaerobic BNF activity

**Figure 3:**
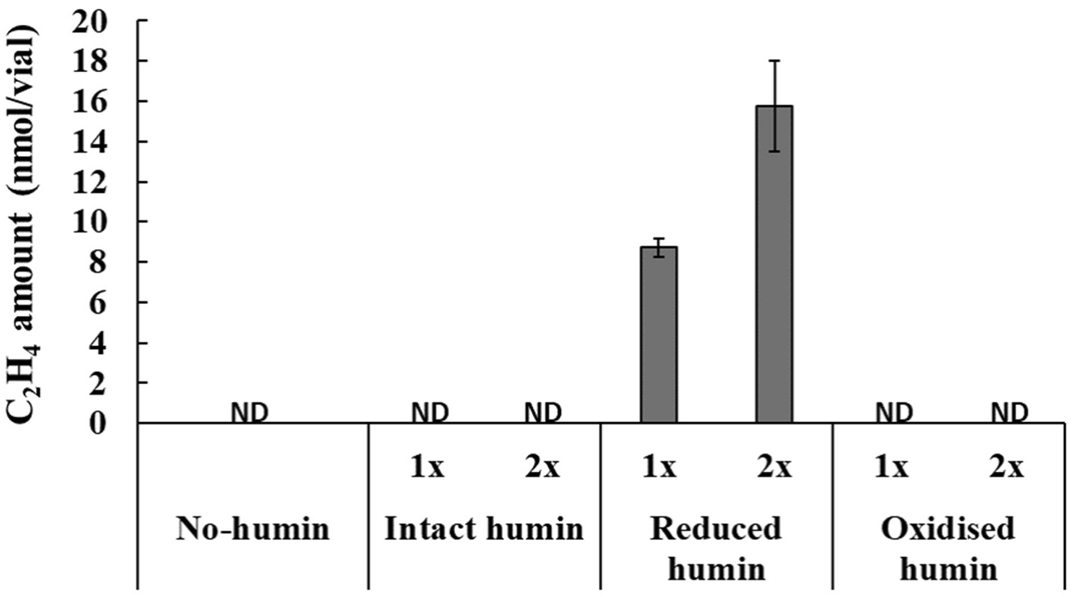
ARA of the no-humin consortium tested with reduced, oxidised and intact humin and without humin. The humin used here was Kamajima humin. Humin concentration was set at two levels as shown by the symbols “1x” and “2x”, indicating 15 g/L and 30 g/L, respectively. *ND* not detected.

**Figure 4:**
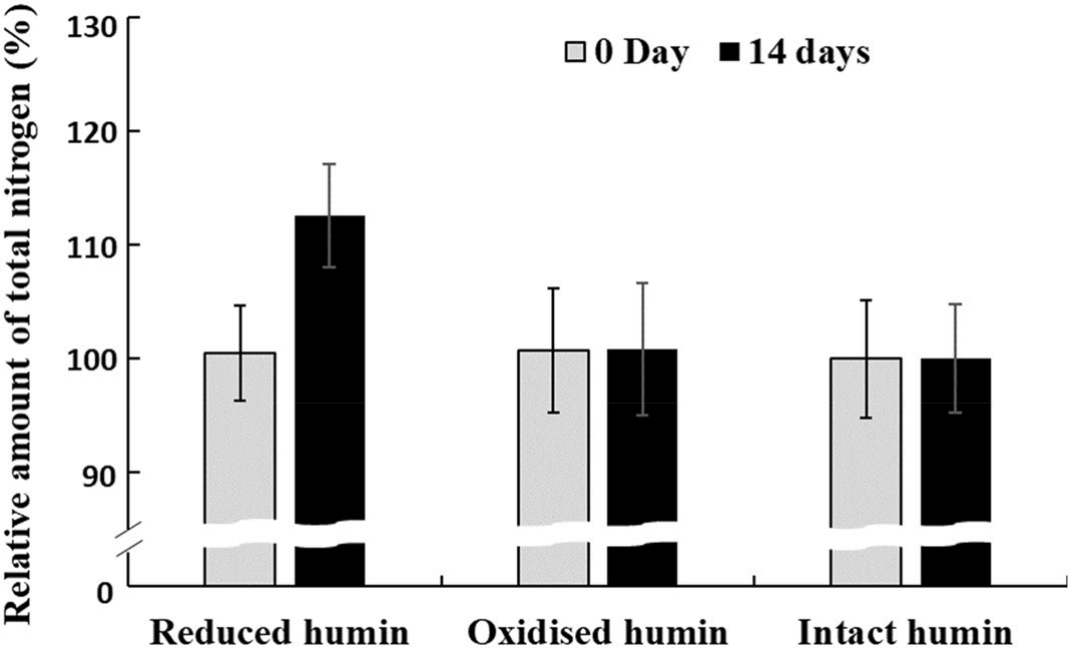
Effect of humin preparation on the nitrogen fixation activity of the no-humin consortium, tested using reduced, oxidised and intact humin, as shown by the change in relative nitrogen content during 14 days of incubation. Gray bars denote the nitrogen contents at 0 day (100%), and black bars denote those after 14 days. The humin used here was Kamajima humin.

The BNF activity of washed and starved no-humin consortium was examined by the ARA test using Helium (He)-bubbled, anaerobic, mannitol-free, MNDA medium supplemented with Kamajima humin in different redox states, in comparison with the no-humin control medium. Figure 3 shows that the consortium tested with the reduced humin only exhibited ARA, which increased two fold, in proportion with the increase in the amount of reduced humin added to the medium. However, the consortium tested with oxidised humin, intact humin, and without humin did not show any activity. The promotion of BNF activity of the consortium was also made apparent in the significant (*P* < 0.05) increase in total nitrogen content in the consortium, but only for the consortium with reduced humin, after 14 days of incubation in the anaerobic mannitol-free MNDA medium at 30 °C (Fig. 4). Total nitrogen content in consortia incubated under other conditions did not increase after the incubation, and remained almost the same as that on day zero.

### Effects of reducing agents on anaerobic BNF activity

**Figure 5:**
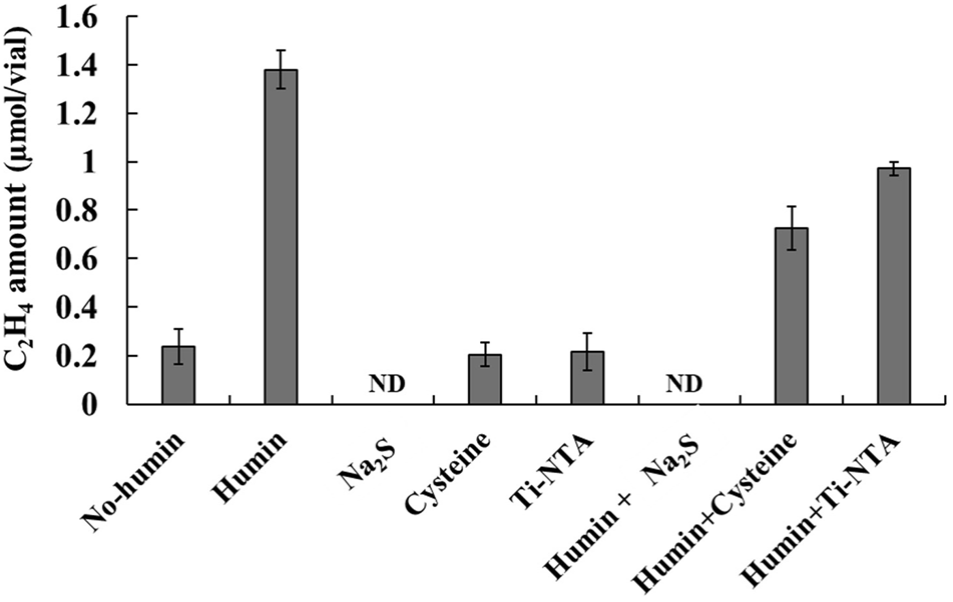
Effect of reducing agents on ARA of the no-humin consortium. The no-humin consortium tested with addition of cysteine or Ti-NTA as reducing agent showed less ARA than the no-humin consortium tested with humin (Kamajima humin, positive control). ARA of the no-humin consortium tested with cysteine or Ti-NTA was increased by the addition of humin, although the value was still lower than the positive control. The reducing agent Na_2_S inhibited ARA of the consortium regardless of the addition of humin. *ND* not detected.

The effect of three commonly used reducing agents (Na_2_S, cysteine, and titanium (III) trinitriloacetate (Ti-NTA)), on the BNF activity of the no-humin consortium was examined by the ARA test in anaerobic MNDA medium, with and without humin. Figure 5 shows that the reducing agents did not promote, but rather inhibited, the BNF activity of the no-humin consortium. The introduction of cysteine and Ti-NTA did not significantly change ARA of the no-humin consortium, while the no-humin consortium tested with humin (positive control) exhibited much higher ARA. The BNF activity of the consortium was higher in the medium with humin in addition to cysteine or Ti-NTA, but still less than that of the positive control. The consortium tested with Na_2_S showed no ARA, regardless of the addition of humin, indicating an inhibitory effect. These findings suggest that the promoting effect of humin on BNF activity differed from that of the reducing agents.

### Effect of micronutrients and vitamins on anaerobic BNF activity

**Figure 6:**
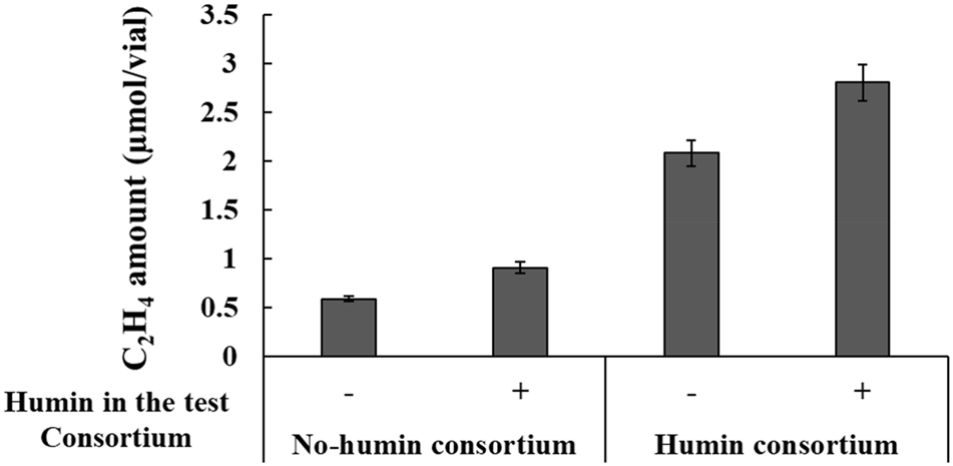
ARA of the no-humin and humin consortia tested in the medium supplemented with micronutrients and vitamins with (+) and without humin (−). The humin used here was Kamajima humin.

The effect of micronutrients and vitamins on BNF activity was examined by the ARA test in anaerobic MNDA medium supplemented with trace element solution SL-10 and vitamin solution under conditions with and without humin. Figure 6 shows that the ARA of the humin consortium and no-humin consortium tested with and without humin under the conditions supplemented with micronutrients and vitamins. Compared with Fig. 1a, the supplementation increased ARA by 52 ± 10% as average of four test conditions. However, the supplementation of micronutrients and vitamins did not change the promoting effect of humin on ARA in the consortia. The addition of humin increased ARA by 77% for the no-humin consortium, and by 40% for the humin consortium, respectively. In comparison between the no-humin consortium tested without humin and the humin consortium with humin, ARA was higher by 5.7 times in the latter. Higher ARA was observed in the humin consortium and under the test conditions with humin (Fig. 6), the same trend as Fig. 1a. These results suggest that humin does not work as a source of micronutrients and vitamins to promote BNF activity. Iron, cobalt, manganese, chromium, zinc and nickel are commonly detected trace elements in humin of different origins^34^. All these trace elements were provided in the medium supplemented with SL-10 solution.

### Microbial community structure

**Figure 7:**
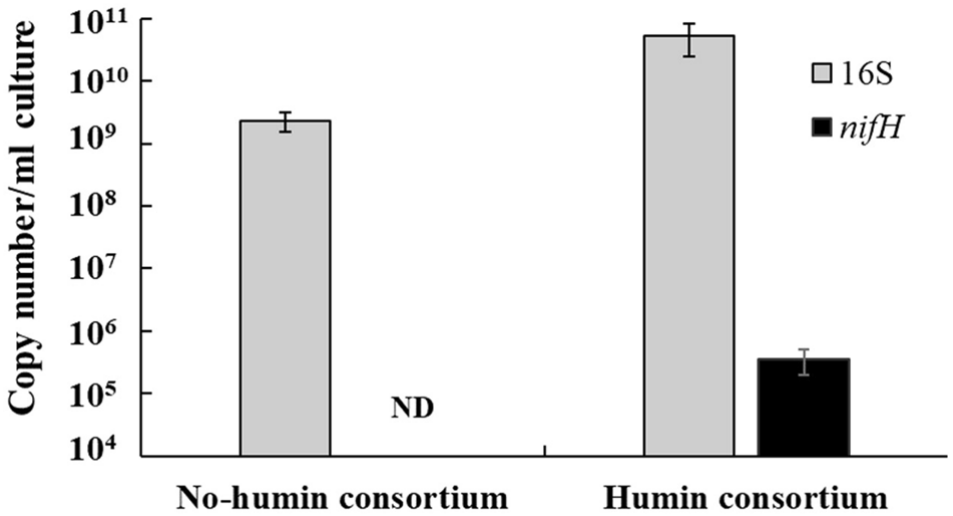
Copy numbers of the 16S rRNA gene (gray bar) and *nifH* gene (black bar) in the no-humin consortium and in the humin consortium in the qPCR. The amplicons of the genes were used to estimate the total and N-fixing population. The primer sets used are described in the materials and methods. *ND* not detected.

Figure 7 shows copy numbers of the 16S rRNA gene, as an indicator of total bacterial population, and of the *nifH* gene, as an indicator of nitrogen-fixing microorganisms, estimated by quantitative PCR (qPCR). Copy numbers of 16S rRNA gene were estimated to exceed 10^9^ copies/ml in both of consortia, although the humin consortium had 100 times larger numbers than the no-humin consortium, indicating the larger microbial population in the humin consortium. The *nifH* gene was only detected in the humin consortium at 10^5^ copies/ml, and not detected in the no-humin consortium. These suggested that nitrogen-fixing microorganisms were not present at a detectable level by the qPCR analysis adopted in the no-humin consortium but were present in the humin consortium. However, the observed small population of nitrogen-fixing microorganisms did not agree with the stable BNF activity detected both in the humin and no-humin consortia following enrichment.

**Figure 8:**
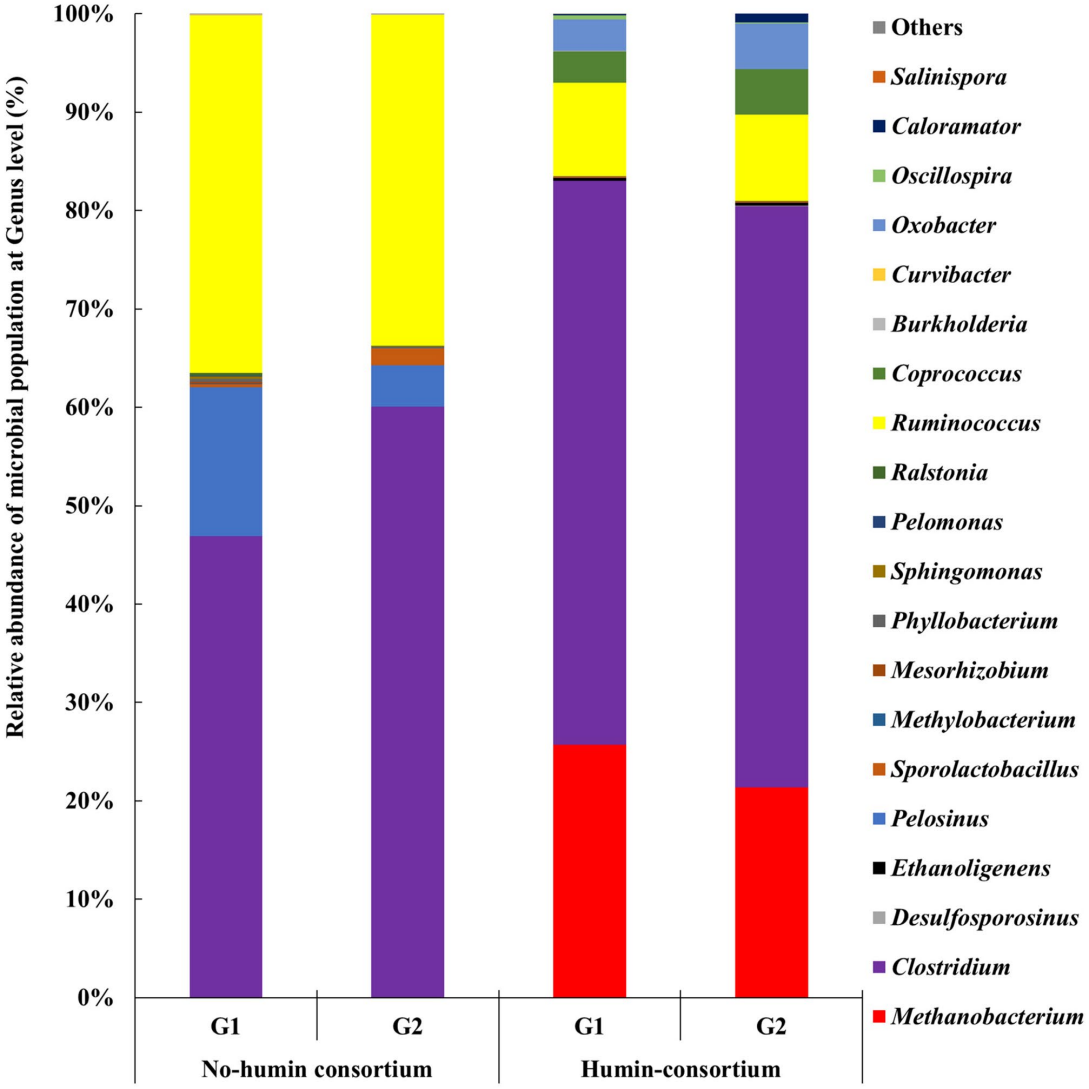
Community structures of the no-humin and humin consortia (35th generation) based on 16S rRNA gene sequencing. The data shows the community structures of two replicates (G1 and G2) of the consortia, individually. “Others” denotes the taxonomic groups with less than 0.02% abundance. In the figure, taxonomic groups are arranged as N-fixers, *nifH*-positive microorganisms and non N-fixers from bottom to top.

The microbial community structures in the humin and no-humin consortia were determined by next generation amplicon sequence of partial 16S rRNA genes using Miseq (Ilmina) (Fig. 8). Bacteria, mostly Firmicutes, accounting for 73–77% of the relative abundance, dominated the humin consortium. *Clostridium, Ruminococcus* and *Oxobacter,* belonging to Clostridiales, were the major genera of detected Firmicutes. Other Clostridiales such as *Desulfocporosinus, Ethanoligenens, Coprococcus, Oscillospira* and *Caloramator* were detected as the minor populations. Other than Clostridiales, the detected genera of Bacteria were *Sporolactobacillus* (Bacillales), *Phyllobacterium* (Rhizobiales), *Sphingomonas* (Sphingomonadales) and *Ralstonia* (Burkholderiales) accounting for several percentage. Archaea, mostly *Methanobacterium,* occupied 22–26% of relative abundance in the humin consortium. The no-humin consortium consisted of mostly Bacteria, in which Firmicutes, mainly *Clostridium, Pelosinus* and *Ruminococcus*, which belong to Clostridiales, occupied over 96% of the relative abundance. The minor populations detected were *Sporolactobacillus* (Bacillales)*, Methylobacterium* (Rhizobiales)*, Mesorhizobium* (Rhizobiales)*,, Phyllobacterium* (Rhizobiales)*, Sphingomonas* (Sphingomonadales), *Pelomonas* (Burkholderiales)*, Ralstonia* (Burkholderiales), *Burkholderia* (Burkholderiales)*, Curvibacter* (Burkholderiales)*,* and *Salinispora* (Burkholderiales). Archaea was present only as negligible abundance, accounted for 0.02% in the no-humin consortium. The same dominant microbial genera, *Clostridium* and *Ruminococcus*, were observed in both the humin and no-humin consortia between the 35th (Fig. 8) and the 11th generation (Fig. S2), consistent with the stable ARA of consortia after the 11th generation (Fig. S1). Among the observed genera of both consortia, *Methanobacterium, Clostridium, Desulfosporosinus, Ethanoligenens, Pelosinus, Sporolactobacillus*, *Methylobacterium, Mesorhizobium, Phyllobacterium, Sphingomonas, Pelomonas* and *Ralstonia* belong to cluster I, cluster II, and cluster III of the N-fixing phylogeny and are known to synthesise nitrogenase, including alternative nitrogenases, for fixing atmospheric N_2_^35–48^. *Ruminococcus, Coprococcus, Burkholderia* and *Curvibacter* have not been reported as N-fixing microorganisms but are known to carry *nifH-like* sequences belonging to Cluster IV of the N-fixing bacterial group^35^. These results suggest that more than 93% of the microbial species, in both consortia, were considered *nifH* positive, contrary to the results of qPCR using *nifH*-specific primers. *Oxobacter, Oscillospira* and *Caloramator* observed in humin consortium, and *Oxobacter* and *Salinispora* observed in no-humin consortium, have not been demonstrated to possess the *nif* gene or BNF activity.

The Shannon diversity index^49^ was 0.50 for the humin consortium, with an evenness of 0.17, and was 1.1 for the no-humin consortium, with an evenness of 0.38. The Chao 1 diversity index^50^, calculated using operational taxonomic units (OTUs), was 19.1 for the humin consortium and 19.3 for the no-humin consortium. These results indicated that although both consortia were enriched with specific microbial species, fewer microbial diversity were present in the humin consortium, than in the no-humin consortium.

## Discussion

Nitrogen fixation in the consortia was promoted with humin. The increase in nitrogen content during 14 days of incubation was significantly higher (by 292%, 11.8 times) in the humin consortium than in the no-humin consortium (Fig. 1b). The nitrogen content in the medium containing humin without inoculation did not show any increase, suggesting no chemical nitrogen fixation by humin. These results suggested that humin promoted the BNF activity of microorganisms. The higher ARA with humin (≥ 40%), regardless of the consortia (Fig. 1a), suggested that the promoting effect of humin was not attributable to the larger population in the humin consortium (Fig. 7) but to increased BNF activity of the microbial cells. This conclusion was also supported by the increased ARA (Fig. 3) and the increase in nitrogen content (Fig. 4) by only reduced humin using the same no-humin consortium. The promotion effect of humin on the BNF activity is also suggested to be distributed widely in the environment, although the extent of the promotion effect would vary, as shown in Fig. 2.

The mechanism of the promotion effect of humin was examined. The promotion of BNF activity was not achieved by the addition of reducing agents, Na_2_S, cysteine, and Ti-NTA, or by the addition of mixtures of the reducing agents and humin (Fig. 5). This indicated that the levels of reduction of the medium did not explain the effect of humin. The promotion of BNF activity by humin was also observed even in anaerobic MNDA medium supplemented with micronutrients and vitamins (Fig. 6), suggesting that the promoting effect of humin was not due to the supply of micronutrients. Comparative studies using electrochemically reduced humin, oxidised humin, and intact humin under the conditions without any other electron donor, demonstrated that only reduced humin promoted BNF activity of the washed non-humin consortium, as shown by ARA (Fig. 3) and the increase in nitrogen content (Fig. 4). No ARA and no increase in nitrogen content were observed with intact and oxidised humin. These results suggested that humin did not serve as organic carbon source donating electrons by the metabolism in cell for the BNF promotion. Rather, humin functioned as extracellular electron mediator for donating electrons to the N-fixing microorganisms. Quinone and sulphur containing moieties have been suggested as redox-active centers of humin^34^. The number of electron equivalents donated by reduced humin was calculated, which promoted the BNF activity of the consortium. The stoichiometry of BNF is given as four electron equivalents required for producing one mole of ammonia from atmospheric N_2_ (Eq. 1). Therefore, the number of electron equivalents donated from humin was estimated to be 236 ± 25 µEq/g-humin for nitrogen fixation, based on the increase of total nitrogen 248 ± 26 µg-N/bottle with 0.3 g of humin (Fig. 4). It can be concluded that intact humin did not serve as organic carbon source but as an extracellular electron mediator in the microbial consortium, thereby promoting BNF, whereby intact humin was reduced by humin-reducing bacteria. This reduced humin subsequently donated electrons to N-fixers of the consortium, resulting in increased BNF activity.

Although the extracellular electron mediating function of humin has been reported for various microbial reactions^30–33^, this is the first study to demonstrate a significant promotion of BNF by humin acting as an extracellular electron mediator. Although the promotion of nitrogen reduction to ammonia using nitrogenase with bioelectrochemical activation^10^ and catalysts or nitrogenase hybrids with photochemical activation^11–15^ has been demonstrated previously, this is the first demonstration of promotion of BNF of whole microbial cells to levels exceeding the biochemical capacity of the cells. The redox potential of half reaction of N_2_/NH_4_^+^ has been reported as − 278 mV (E^0^′)^52^. The viologens, artificial mediators useful for donating electrons to nitrogenase, have been reported to require a redox potential lower than  − 360 mV (*versus* a normal hydrogen electrode) to be effective^53^. Such low redox potential of humin have been suggested in CO_2_-reducing acetogenesis assisted by humin (− 290 mV, E^0^′ of CO_2_/CH_3_COOH)^54^. The isoelectric potential of humin has been also reported ranging from − 400 to + 272 mV (*versus* standard hydrogen electrode) in the cyclic voltammetry analysis of humin^34^. These suggested that humin has redox active moieties with lower (more negative) redox potential, which is thermodynamically feasible for nitrogen fixing reaction.

In addition to the BNF promotion, the previous reports also demonstrated that humin promoted denitrification^33^ and nitrate reduction to ammonia^32^ as extracellular electron mediator. Humic substances (natural organic matter) have been also shown to serve as terminal electron acceptors to fuel anammox reaction (anaerobic ammonium oxidation to N_2_)^51^. Given the wide distribution of humic substances including humin, it is suggested that humin and/or humic substances would play a vital role for global nitrogen cycle under anaerobic environments, not only promoting BNF but also nitrogen loss to atmosphere.

The analysis of community structures demonstrated that both consortia were dominated by *Clostridium* (Fig. 8). *Clostridium* is an anaerobic N-fixer. The promotion of BNF by humin in both consortia could be attributed to extracellular electron donation from humin to *Clostridium*. *Methanobacterium* (Archaea) was only abundant in the humin consortium. *Methanobacterium,* an anaerobic N-fixer as well as hydrogenotrophic methanogen^55^, may obtain the extracellular electrons from humin, resulting in the propagation in the humin consortium. *Ethanoligenens* and *Desulfosporosinus* were detected only in the humin consortium although they are minor populations (0.35%), suggesting that they are humin-dependent microorganisms. *Ethanoligenens* and *Desulfosporosinus* were reported to fix nitrogen using an alternative nitrogenase^44^. The roles of *Ruminococcus*, *Coprococcus* and *Curvibacter* remain unknown because they carry *nifH*-like genes belonging to Cluster IV, which are not involved in nitrogen fixation^35^.

More than 93% genera in both consortia harbour the *nifH* gene^35,36,41–44,56^. However, qPCR analysis (Fig. 7) demonstrated that the N-fixing microbial population was significantly smaller (10^–5^) than that of the total microbial population in the humin consortium and even not detected in the no-humin consortium. Although qPCR assays using different primer sets (F2/ R6, PolF/PolR, and IGK /NifH3)^57^ were undertaken to quantify the *nifH* genes, zero, or poor, amplification was observed in all attempts (Fig. S3). This could be due to the low sensitivity of these primer sets for *nifH* genes of the consortia. In preliminary trials to isolate anaerobic microorganisms from the consortia, 18 cultures were all positive in ARA (data not shown), suggesting a dominance of N-fixing microorganisms in the consortia. Further studies should be undertaken to identify the isolates and *nif* genes. *Oxobacter, Caloramator,* and *Oscillospira* without N-fixing ability in the humin consortium indicated the availability of N sources other than nitrogen gas for growth. An increase in ammoniacal nitrogen concentration during the incubation was observed in both consortia, which would be released as excess NH_3_ by the N-fixers in the community (Fig. S4).

In conclusion, this study demonstrated that humin promoted the BNF activity of N-fixing anaerobic consortia by 5.7–11.8 times as shown by the increases in ARA and the nitrogen content. Humin did not function as a reducing agent or as a source of micronutrients or vitamins, or as organic carbon source, but as an extracellular electron mediator to donate electrons to N-fixing consortia. The consortium enriched with humin was dominated by *Clostridium*, *Ruminococcus*, and *Methanobacterium*, while the consortium enriched without humin was dominated by *Clostridium*, *Ruminococcus*, and *Pelosinus* as possible N-fixers. Further studies are needed to identify humin-promoted specific N-fixers. Moreover, the mechanism of the electron mediating function of humin for N-fixers is still under study. However, considering the promotion of BNF activity under nitrogen-deficient conditions and the ubiquitous distribution of humin, the implementation of humin-promoted atmospheric N-fixation would contribute to the development of a sustainable technology. In addition, considering the promoting effects of humin on denitrification and nitrate reduction to ammonia, humin would be playing an important role for global N-cycle, especially under anaerobic environment.

## Materials and methods

### Humin preparation

Humin was extracted from the following surface soils or sediments: Kamajima paddy soil, Nagoya University farm upland soil, Ibakaki upland soil, Yatomi paddy soil, and Arako river sediment, as previously reported^58^, with some modifications. Briefly, the soils and sediment were air-dried and sieved through 1 mm mesh. The sieved soils or sediment (100 g) were repeatedly washed by shaking with a solution (24 h; compositions defined below), centrifugation (8000 *g*, 15 min, 24 °C), followed by decantation. The washing chemical solutions were as follows: ten times with 150 mL of 2% HF, ten times with 150 mL of 0.1 M NaOH, and twenty times with 150 mL of ultrapure water. After washing, pH was adjusted to 7.5 using 0.1 M NaOH. The pH-adjusted humin was freeze-dried, ground using a ceramic mortar and pestle, and then used experimentally. The C, H, and N contents of humin were determined using a CHN analyser (Yanaco MT-5 CHN-corder, Yanaco New Science Inc., Kyoto, Japan) and the results are shown in Table S1.

### Enrichment of humin and no-humin consortia with nitrogen-fixing activity

Kamajima paddy soil was collected on 6th January 2009 and used as an inoculum. 250 mL of a modified nitrogen-deficient Ashby (MNDA) medium^59^ and 150 mL of water-saturated soil was mixed in a 600 ml glass bottle and subsequently sealed with a butyl rubber stopper and aluminium cap. The headspace was flushed with nitrogen gas to achieve anaerobic conditions. The MNDA medium was composed of mannitol (20 g/L), K_2_HPO_4_ (0.2 g/L), MgSO_4_·7H_2_O (0.2 g/L), NaCl (0.2 g/L), K_2_SO_4_ (0.1 g/L), and CaCO_3_ (5 g/L). After seven weeks of incubation at 30 °C under static conditions, 2 mL of the culture was aseptically transferred to a 50 mL-volume glass vial containing 20 mL of anaerobic MNDA medium. A supplement of 15 g/L of humin was added prior to the preparation of anaerobic MNDA medium in the vial. Thus, two anaerobic consortia were enriched: one using the anaerobic MNDA medium supplemented with humin (humin consortium) and another using the same medium but without humin (no-humin consortium). Transfer occurred once every four weeks until the 6th generation, and then reduced to every two weeks to increase the frequency of culture transfer. The culture transfer was maintained to the 11th generation to obtain enriched N-fixing microbial consortia with stable ARA. The BNF activity of the consortia was examined using either ARA or the increase in nitrogen in the culture.

### Acetylene reduction activity test

ARA is the widely used indicator of the biological N-fixation activity of diazotrophs^60,61^. Therefore, the ARA test was undertaken to examine nitrogen fixation activity indirectly because acetylene is reduced to ethylene by nitrogenase, which is also responsible for BNF^62^. The ARA test was undertaken as follows. Two sets of 10 ml-volume glass vials (one set supplemented with 0.03 g of humin in each vial, another set having no humin) were flushed with helium gas, then, added aseptically with 2 mL of the He-bubbled anaerobic MNDA medium. After that, 2 mL of inoculum from the humin-consortium and no-humin consortium was separately inoculated into each of the two sets of the vials. Finally, all the vials were spiked with 200 µL of acetylene gas and incubated under static conditions at 30 °C for one week. After incubation, 100 µL of the gaseous sample was drawn from the headspace using a Pressure-Lok^R^, VICI Precision Analytical Syringe (Baton Rouge, LA USA). The concentrations of acetylene and ethylene in the sample were determined using a GC-14B gas chromatograph (Shimadzu, Kyoto, Japan) equipped with a Molecular Sieve-5A column (60/80 mesh and 3 m in length) and a flame ionisation detector. Nitrogen gas was used as the carrier gas at a flow rate of 60 mL/min. H_2_ gas and compressed air were provided for the detector at flow rates of 50 mL/min and 450 mL/min, respectively. The temperatures of the column, injector, and detector were 80 °C, 100 °C, and 100 °C, respectively.

### Determination of nitrogen concentration

Biological nitrogen fixation activity was determined directly by measuring the increase in concentration of nitrogen in the culture. For this determination, the individual consortia were sealed in a 50 mL vial containing 30 mL of the anaerobic MNDA medium with or without the supplementation of humin (15 g/L), bubbled with nitrogen gas for 90 min, and the headspace was flushed for 5 min. The medium was inoculated aseptically with 3 mL of one of the consortia. Sampling was undertaken by taking 10 mL of the consortium using a syringe prior to incubation. After two weeks of incubation at 30 °C, an additional 10 mL of the consortium was collected in the same manner. These samples were transferred to a ceramic mortar and dried at 105 °C for two days. Dried samples were ground and approximately 2000 µg of sample used for the determination of nitrogen content. The elemental composition of C, H, and N of dried samples was determined using a CHN analyser. All measurements were replicated a minimum of three times.

### Effects of various humins on ARA

The effects of various humins on BNF activity of the no-humin consortium were examined using the ARA test. Humin sources were as follows: Kamajima humin, Nagoya University farm (NU farm) humin, Ibakaki humin, Yatomi humin, and Arako humin. The ARA test of the no-humin consortium without the addition of humin was provided as the control. The effects were evaluated using triplicate experiments.

### Effects of reducing agents on ARA

Effects of reducing agents on N-fixation activity of the no-humin consortium were examined by the addition of different reducing agents, including 15 mM Na_2_S, 1.7 mM L-cysteine hydrochloride, 0.025 mM titanium (III) trinitriloacetate-(Ti-NTA), which were added to the anaerobic MNDA medium, with and without the supplementation of Kamajima humin (15 g/L). ARA tests were conducted at least three times.

### Effect of micronutrients and vitamins on ARA

The effects of micronutrients and vitamins on ARA were examined because the anaerobic MNDA medium did not contain micronutrients and vitamins. Micronutrients and vitamins were added as 1 ml of trace element SL-10 solution^63^ and 10 mL of vitamin solution^64^ to 1 L of the anaerobic MNDA medium to make the improved Ashby medium. The ARA test was then performed using the no-humin consortium in the improved Ashby medium with and without supplementation of Kamajima humin (15 g/L).

### Effect of humin prepared under oxidised and reduced conditions on BNF

The effects of different redox state of humin on the BNF activity of the no-humin consortium were examined experimentally. Reduced and oxidised Kamajima humin were prepared in 200 mL of 0.5 M NaCl solution using an electrochemical system under anaerobic conditions, in a vinyl anaerobic chamber equipped with a vacuum airlock and two glove ports (Coy-7450000, COY, Grass Lake, MI, USA). The electrochemical system consisted of a potentiostat (Automatic Polarisation System HSV-110, Hokuto Denko, Osaka, Japan), two twisted platinum electrodes (0.8 mm in diameter and 1 m in length) as working and counter electrodes, and a Ag/AgCl reference electrode (+ 0.199 V *versus* the standard hydrogen electrode (SHE)). Redox potential was maintained at − 0.4 V or + 0.4 V (*versus* SHE) for 24 h to reduce or oxidise 2 g of autoclaved humin, respectively. After equilibrium was attained, the oxidised or reduced humin was collected by filtration and dried using a vacuum pump. In this test, the washed and starved non-humin consortium was used. The non-humin consortium was incubated at 30 °C for 2 weeks in anaerobic MNDA medium in which the buffer CaCO_3_ was replaced with 30 mM HEPES (anaerobic MNDA HEPES medium). After incubation, the consortium was washed three times by suspending it in mannitol-free anaerobic MNDA HEPES medium and by decanting the supernatant after centrifuging the consortium at 2500×*g* for 5 min at 4 °C. The washed consortium was then kept for starvation in the mannitol-free anaerobic MNDA medium for two days. After washing and starvation pre-treatments, the no-humin consortium was subjected to a test evaluating the effect of different redox states of humin on BNF activity. Biological nitrogen fixation activity was evaluated using both the ARA test with He-bubbled, organic carbon source-free (mannitol-free), anaerobic MNDA medium and the increase in nitrogen content using N_2_-bubbled, organic carbon source-free (mannitol-free), anaerobic MNDA medium, by incubating at 30 °C under static condition for two weeks. All tests were conducted a minimum of three times.

### qPCR for total and nitrogen-fixing bacteria in the consortia

Microbial DNA was extracted using a FastDNA SPIN kit for soil (MP Biomedicals, Santa Ana, CA, USA) according to the manufacturer’s instructions. Populations of total and N-fixing bacteria in the consortia were estimated by qPCR using a universal primer set targeting the 16S rRNA gene (27F: 5′-AGAGTTTGATCCTGGCTCAG-3′, and 1492R: 5′-GGTTACCTTGTTACGACTT-3′)^65^ and specific primer set targeting the *nifH* gene, PolF and PolR. PolF was the forward primer ( 5′-TGCGAYCCSAARGCBGACTC-3′: Y = C and T; S = C and G; R = A and G; B = G, T, and C) and PolR the reverse primer (5′-ATSGCCATCATYTCRCCGGA-3′: S = C and G; Y = C and T; R = A and G)^57^. qPCR was performed using the LightCycler system (Roche Diagnostics, Basel, Switzerland). The reaction mixtures (final volume 20 µL) contained 12.4 µL PCR grade H_2_O, 1.6 µL of 25 mM MgCl_2_, 1 µL of 10 μM forward primer, 1 µL of 10 μM reverse primer, 2 µL LightCycler FastStart DNA Master SYBR Green I (Roche Molecular Biochemicals, Mannheim, Germany) and 2 µL template microbial DNA. To generate a standard curve, DNA fragments of 16S rRNA and *nifH* genes were amplified by PCR using Ex Taq polymerase (TaKaRa Bio Inc., Kusatsu, Shiga, Japan), and the PCR products were purified using a QIAEX II Gel Extraction Kit (Qiagen, Düsseldorf, Germany) according to the manufacturer’s instructions. A dilution series of the purified DNA fragment of each gene was amplified to generate a standard curve. The PCR cycling conditions consisted of a pre-incubation step at 95 °C for 10 min followed by a quantification step consisting of 45 cycles of denaturation at 95 °C for 10 s, annealing at 55 °C for 10 s, and elongation at 72 °C for 10 s. After amplification, the melting curve was obtained by heating the PCR products at 95 °C, followed by cooling at 65 °C for 15 s and then gradually increasing the temperature to 95 °C at the rate of 0.1 °C/ s. Cooling was undertaken at 40 °C for 30 s.

### Analysis of community structure in consortia

The partial 16S rRNA amplicon sequence was performed for the DNA fragments obtained by PCR amplification with the primer set targeting the V3-V4 region: Pro341F (5′- CCT ACG GGN BGC ASC AG-3′) and Pro805R (5′- GAC TAC NVG GGT ATC TAA TCC-3′)^66^. The PCR mixtures contained 5 µL of template DNA (5 ng/µL), 2.5 µL of Pro341F and Pro805R primers (2 µM each), and 12.5 µL of KAPA HiFi HotStart Ready mix (KAPA Biosystems, Wilmington, MA, USA). The PCR reaction was performed as follows: initial activation at 94 °C for 30 s, followed by 10 cycles at 94 °C for 10 s, 60 °C for 30 s, 72 °C for 30 s, followed by 10 cycles at 94 °C for 10 s, 59 °C for 30 s, 72 °C for 30 s, followed by 10 cycles at 94 °C for 10 s, 58 °C for 30 s, 72 °C for 30 s, and a final extension at 72 °C for 4 min. Purification of the PCR products was carried out using the AMPure XP kit (Beckman Coulter Genomics Inc., Brea, CA, USA) according to the manufacturer’s instructions, and the PCR products were confirmed using 1% agarose gel electrophoresis. The concentration of purified DNA was determined using a QuantiFluor dsDNA System (Promega Corporation, Fitchburg, WI, USA), and sequencing of the purified DNA was performed using a Miseq platform with a Miseq reagent kit v3 (600 cycle, Illumina Inc., San Diego, CA, USA). A chimaera check for the base sequences of each read obtained from the analysis was carried out using USEARCH v6.1^67^. Sequence reads with more than 97% similarity were classified into the same operational taxonomic unit (OTU), and OTU picking, and a cluster analysis was performed in QIIME 2^68^. Finally, OTUs were identified using the Green-gene database (ver. 13_8) as a reference^69^.

## Supplementary Information

Supplementary Information.
