# Supplementary material for: Promotion of biological nitrogen fixation activity of an anaerobic consortium using humin as an extracellular electron mediator
Source: Sci Rep. 2021 Mar 22;11:6567. doi: 10.1038/s41598-021-85955-3 (PMC7985497; doi:10.1038/s41598-021-85955-3)
Supplement: Supplementary file 1 — Supplementary Information. [file 41598_2021_85955_MOESM1_ESM.pdf]

Supplementary Information

Promotion of biological nitrogen fixation activity of an anaerobic consortium using humin as an extracellular electron mediator

Sujan Dey[1]*, Takanori Awata[2]*, Jumpei Mitsushita[1], Dongdong Zhang[3], Takuya Kasai[1,3], Norihisa Matsuura[4], Arata Katayama[1,3]*****

[1] *Graduate School of Engineering, Nagoya University, Chikusa-ku, Nagoya 464-8603, Japan*

[2] *National Institute for Land and Infrastructure Management, Asahi 1, Tsukuba, Ibaraki 305-0804, Japan*

[3] *Institute of Materials and Systems for Sustainability, Nagoya University, Chikusa-ku, Nagoya 464-8603, Japan*

[4] *School of Geosciences and Civil Engineering, Kanazawa University, Kakuma-machi, Kanazawa, Ishikawa 920-1192, Japan*

*These authors contributed equally as the first authors to this study.

** Present address: Ocean College, Zhejiang University, Zhoushan, 316021, China

Short running title: **Promotion of biological nitrogen fixation**

***Corresponding author:

Arata Katayama

Tel: +81-52-789-5856

Fax: +81-52-789-5857

E-mail: katayama.arata@nagoya-u.jp

a)

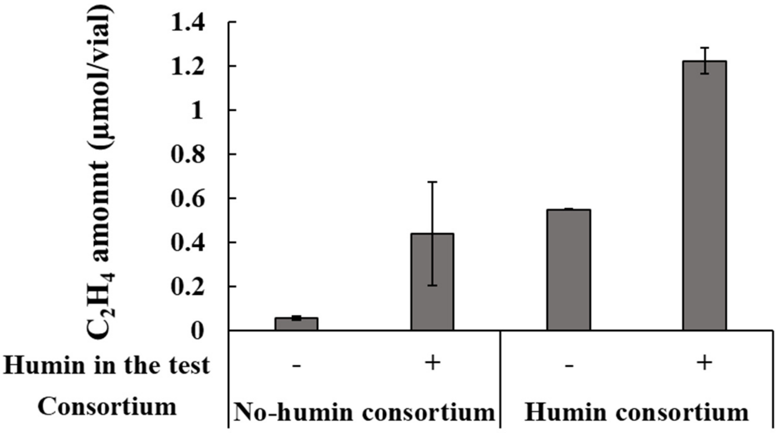

b)

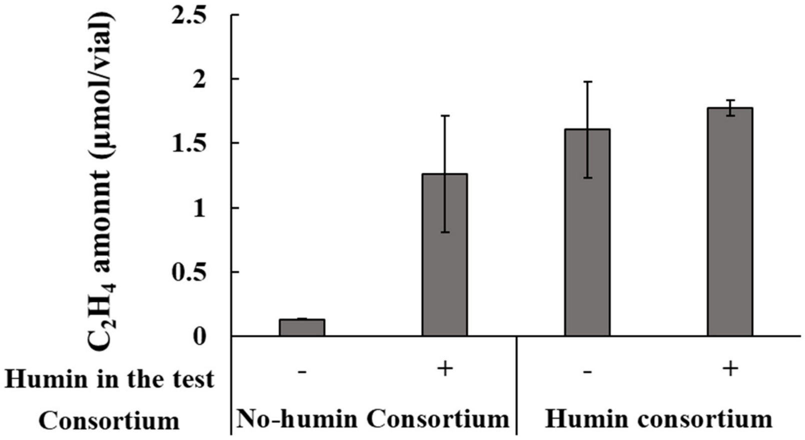

c)

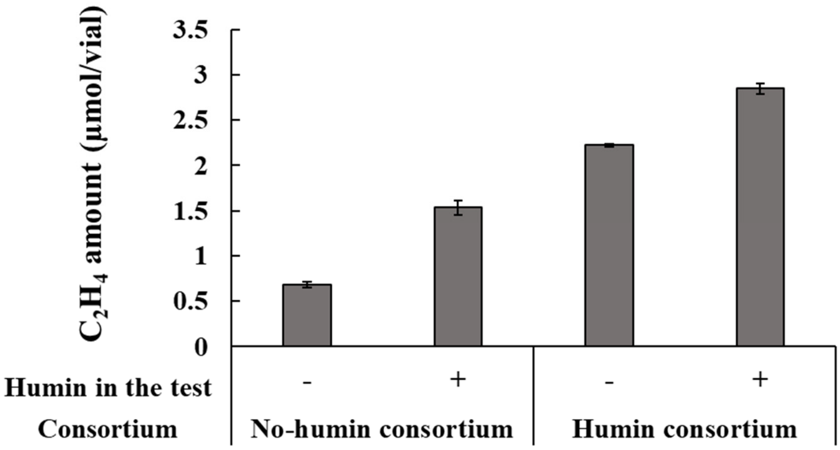

Supplementary Figure S1. Stable ARA of the humin and no-humin consortia of different culture generations. (a) generation 11; (b) generation 15; (c) generation 60) with (+) and without humin (-). The humin used here was Kamajima humin.

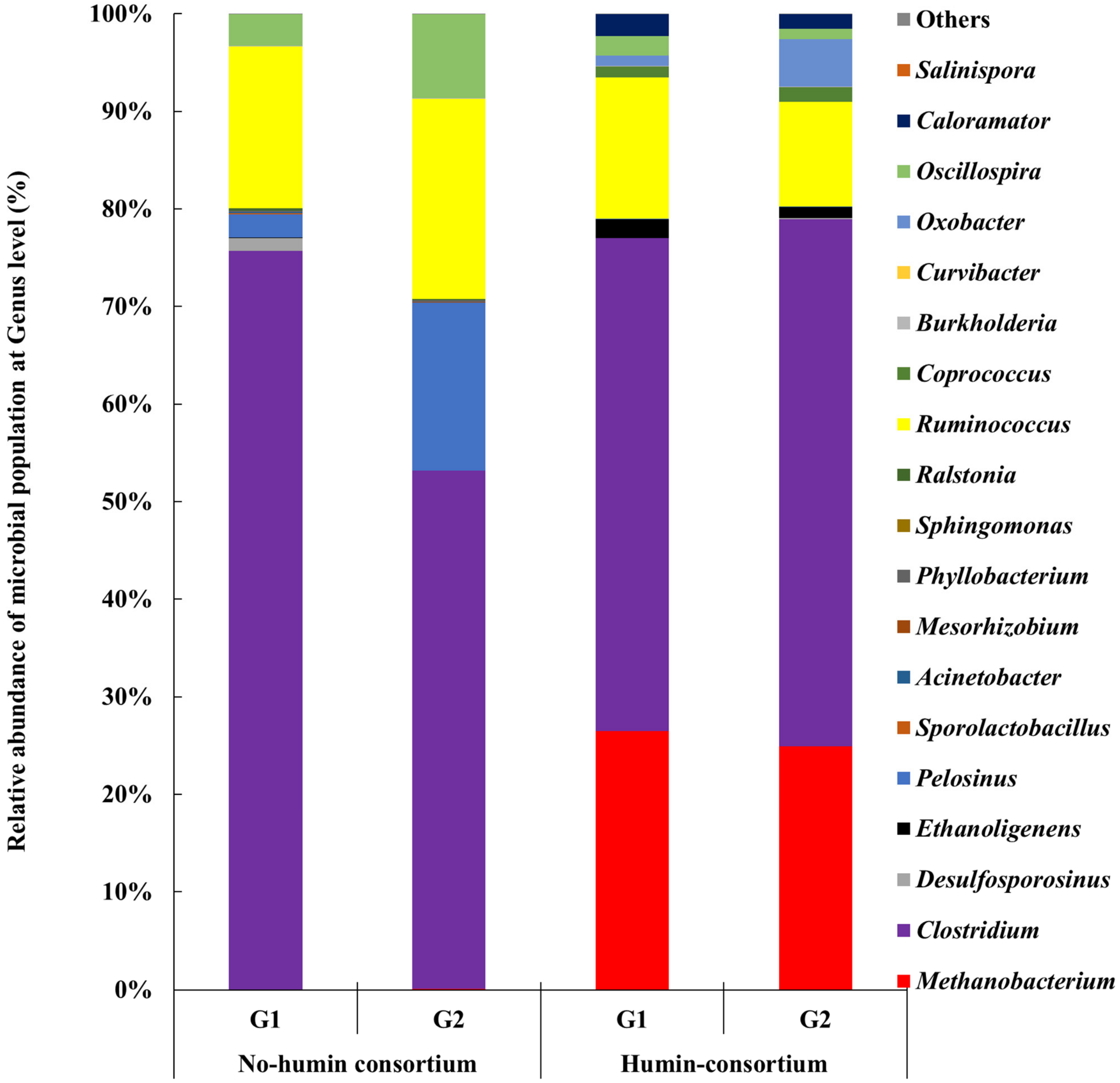

Supplementary Figure S2: Community structures of the no-humin and humin consortia (11th generation) based on 16S rRNA gene sequencing. The data shows the community structures of two replicates (G1 and G2) of the consortia, individually. “Others” denotes the taxonomic groups with less than 0.02% abundance. In the figure, taxonomic groups have been arranged as N-fixers, *nifH* positive microorganisms and non N-fixers from bottom to top.

a)

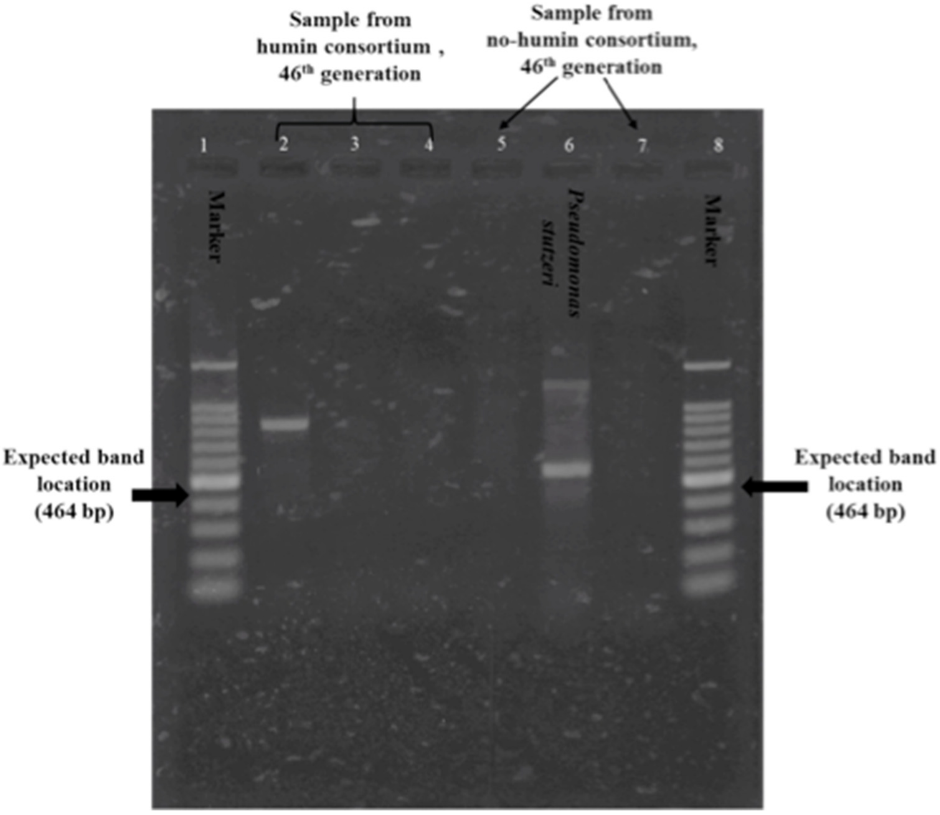
Sample from
humin consortium ,
46th generation
Sample from
no-humin consortium,
46th generation
1
2
3
4
5
6
7
8
Marker
Pseudomonas
stutzeri
Marker
Expected band
location
(464 bp)
Expected band
location
(464 bp)

b)

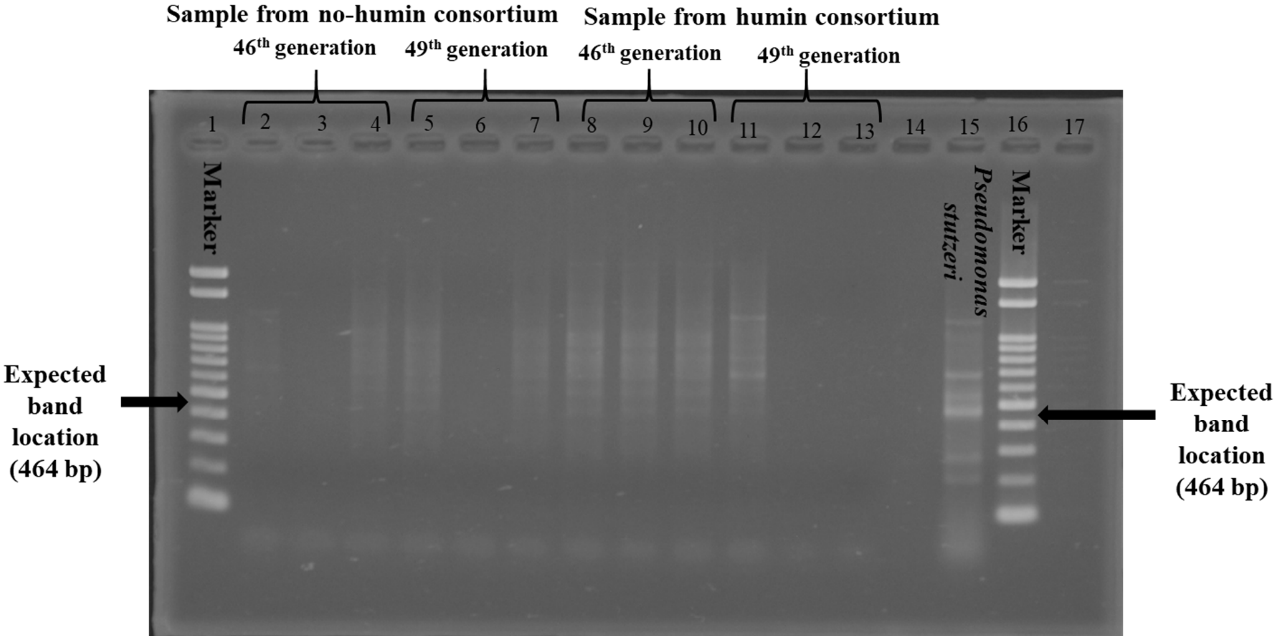
Sample from no-humin consortium
46th generation
49th generation
Sample from humin consortium
46th generation
49th generation
1
2
3
4
5
6
7
8
9
10
11
12
13
14
15
16
17
Marker
Pseudomonas
stutzeri
Marker
Expected
band
location
(464 bp)
Expected
band
location
(464 bp)

c)

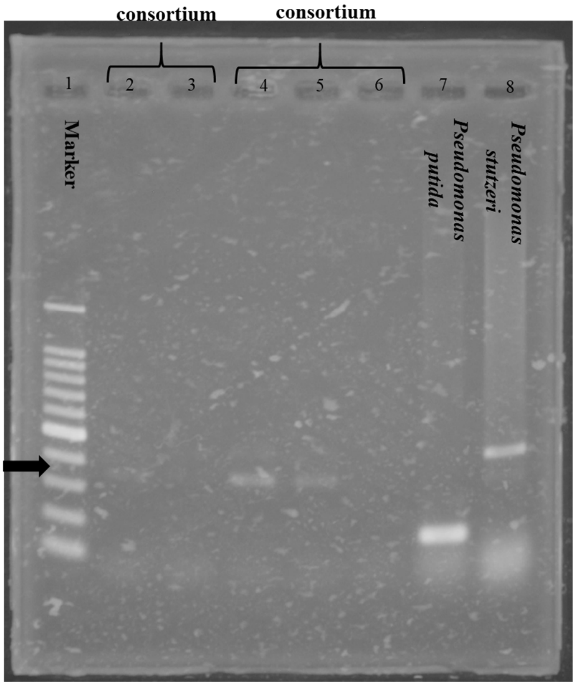
Sample from no-humin consortium
Sample from humin consortium
1
2
3
4
5
6
7
8
Marker
Pseudomonas putida
Pseudomonas stutzeri
Expected band location (359 bp)

d)

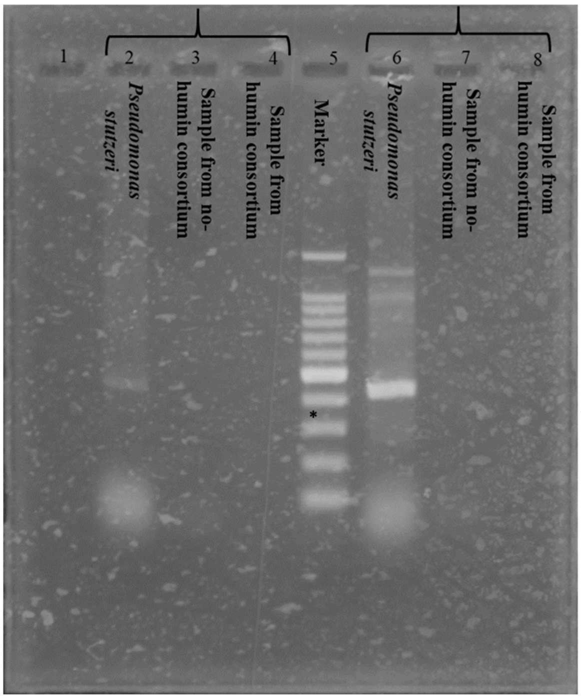
Annealing temperature 55 °C
Annealing temperature 48 °C
1
2
3
4
5
6
7
8
Pseudomonas stutzeri
Sample from no-humin consortium
Sample from humin consortium
Marker
Pseudomonas stutzeri
Sample from no-humin consortium
Sample from humin consortium
*
* Expected band location (359 bp)

Supplementary Figure S3: PCR amplification of the *nifH* gene of the microbial consortia using two different sets of primer pairs: a) IGK/nifH3[1] (Forward primer IGK: 5'-AARGGNGGNATHGGNAA -3', Reverse primer nifH3: 5'-ATRTTRTTNGCNGCRTA-3') with annealing temperature at 52°C. Three DNA samples extracted from humin consortium and two DNA samples from no-humin consortium, both 46th generation, were amplified using IGK/nifH3 primer pair with DNA samples from *Pseudomonas stutzeri* (positive control for *nifH* gene). All the DNA extraction was carried out using a FastDNA SPIN kit for soil (MP Biomedicals, Santa Ana, CA, USA) according to the manufacturer's instructions, and the samples containing 20 ng/µl - 25 ng/µl DNA were used for the PCR reaction. The PCR amplification for the *nifH* gene was carried out as follows: Pre-incubation 94°C 1 min, 30 cycle of denaturation at 98°C for 10 sec, annealing at 52°C for 30 sec, elongation at 72°C for 30 sec and final elongation 72°C for 5 mins. The PCR reaction mixture, 20 µl as total, consisted of 1 µl of template DNA, 0.5 µl of 50 µM forward primer, 0.5 µl of 50 µM reverse primer, 2 µl of 10X Ex taq buffer, 2 µl of 10mM dNTP mixture, 0.2 µl of Ex taq polymerase, and 13.8 µl of PCR grade water[1]. After the PCR reaction, 5µl of the PCR products were loaded individually and subjected to agarose gel electrophoresis. The electrophoresis was performed using a Mupid®-2x submarine electrophoresis system (Mupid Co., LTD, Tokyo, Japan). Then, the gel was treated with ethidium bromide solution for 15 minutes, and the DNA bands were visualized using a high performance UV transilluminator (DigiDoc-it® 125 imaging system and UVPDoc-it® LS image acquisition software, version: 7.1 RC 3.38, Analytik Jena US LLC, Upland, CA, USA). Marker DNA ladder was electrophorated together, showing the bands at the range of 100 base pair (bp) to 1500 bp at 100 bp intervals (TaKaRa Bio, Kusatsu, Shiga, Japan). The positive control showed the amplified DNA band. However, except for one sample from humin consortium, the DNA samples from humin and no-humin consortia did not show the amplified DNA band. **b) IGK/nifH3[1] (Forward primer IGK: 5'-AARGGNGGNATHGGNAA -3', Reverse primer nifH3: 5'-ATRTTRTTNGCNGCRTA-3') with annealing temperature at 47°C.** Six DNA samples extracted from humin consortium and six DNA samples from no-humin consortium, from two different generations (46th and 49th generations), were amplified at the same time using IGK/nifH3 primer pair in the same manner as described above except for the annealing temperature at 47°C. In the electrophoresis, the marker DNA ladder was changed to the one with bands at the range of 100 bp to 2000 bp at 100 bp intervals (Nippon gene Co. LD. Toiya-machi, Toyama, Japan). Although the lower annealing temperature was used, there was no or very weak amplified DNA bands observed in both humin and no-humin consortia. While, the positive control, the sample from *Pseudomonas stutzeri*, showed the amplified DNA band at

the expected location. With the results of gel a), the presence of *nifH* gene was not confirmed in both humin and no-humin consortia by the PCR reaction using IGK/nifH3 primer pairs. No sample was loaded in the lanes No. 14 and 17. **c) F2/R6[1] (Forward primer F2: 5'- TGYGAYCCIAAIGCIGA-3', Reverse primer R6: 5'- TCIGGIGARATGATGGC-3').** Two DNA samples extracted from no-humin consortium and three DNA samples from humin consortium, both 46th generation, were amplified using F2/R6 primer pair, with the DNA templates from *Pseudomonas stutzeri* and *Pseudomonas putida* as positive controls for *nifH* genes. The PCR amplification of *nifH* gene using F2/R6 primer pair was carried out as follows: Pre-incubation 94°C 1 min, 30 cycle of denaturation at 98°C for 10 sec, annealing at 51°C for 30 sec, elongation at 72°C for 30 sec and final elongation 72°C for 5 mins. The DNA sample preparation and the composition of the PCR reaction mixture were the same as the ones of the PCR amplification with IGK/nifH3 primer pair, described above. After the PCR reaction, the agarose gel electrophoresis and DNA band visualization were carried out in the same way as explained for the gel a). There were amplified DNA bands observed in one out of two samples in no-humin consortium and two out of three samples from humin consortium, although the bands were weak compared with the positive controls. **d) F2/R6[1] (Forward primer F2: 5'- TGYGAYCCIAAIGCIGA-3', Reverse primer R6: 5'- TCIGGIGARATGATGGC-3') with annealing temperature at 55°C and 48°C.** One DNA sample of humin consortium and one DNA sample of no-humin consortium, both 46th generation, were amplified using F2/R6 primer pair by using different annealing temperature, 55°C (for the lanes 2, 3 and 4) and 48°C (for the lanes 6, 7 and 8). All the other conditions and procedures were the same as the PCR amplification explained for the gel c). No amplified product was observed in either annealing temperature, while the positive control showed the amplified DNA bands. Therefore, the *nifH* gene detection was not conclusive in the PCR amplification using F2/R6 primer pair. No sample was loaded in the lane No.1. **Abbreviation: Y = C and T; S = C and G; R = A and G; B = G, T and C; H= A, C, T; N= A, T, G, and C; I = Inosine.**

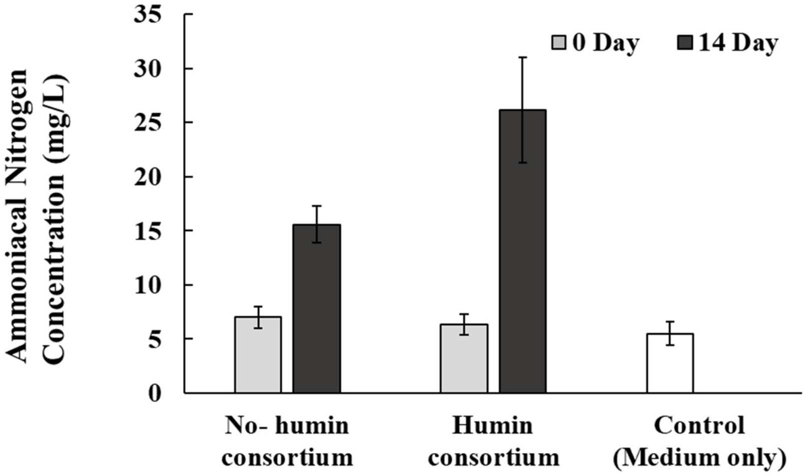

Supplementary Figure S4: Ammoniacal nitrogen (NH_4^+/NH_3) concentration of the no-humin and humin consortium. Black bars represent the ammonia concentration on day 14, gray bars represents the ammonia concentration on day 0, white bars represent the ammonia concentration of the media. Ammoniacal nitrogen concentration was determined using a Metrohm 761compact ion chromatograph (Herisau, Switzerland). The detection limit of ammoniacal nitrogen was 7.33 µM. The determination was carried out in both humin and no-humin consortia before (0 day) and after (14 day) incubation under static condition at 30°C.

Supplementary Table S1: Composition of carbon (C) , hydrogen (H), and nitrogen (N) of different humins analysed by CHN ash analysis.

Humin	C	H	N	C/N	H/C
**Kamajima**	0.37	0.25	0	----	8.11
**Nagoya University farm**	18.98	2.23	2.31	9.59	1.41
**Arako**	9.48	1.42	0.6	18.43	1.80
**Ibaraki**	4.26	0.97	0.39	12.74	2.73
**Yatomi**	2.56	1.02	0.15	19.91	4.78

References

1. Gaby, J. C. & Buckley, D. H. A comprehensive evaluation of PCR primers to amplify the nifH gene of nitrogenase. *PLoS One* **7**, (2012).
